# Update on Prenatal Detection Rate of Critical Congenital Heart Disease Before and During the COVID-19 Pandemic

**DOI:** 10.1007/s00246-024-03487-9

**Published:** 2024-04-03

**Authors:** Deepak Gupta, Tiffany Vuong, Shuo Wang, Lisa M. Korst, Jay D. Pruetz

**Affiliations:** 1 Children’s Hospital Los Angeles, Department of Pediatrics, Los Angeles, California USA; 2 Keck School of Medicine of USC, Los Angeles, California USA; 3 Children’s Hospital Los Angeles, Division of Cardiology, Keck School of Medicine of USC, Los Angeles, California USA; 4 Childbirth Research Associates, LLC, North Hollywood, Los Angeles, California USA; 5 Children’s Hospital Los Angeles, Division of Cardiology, Fetal Cardiology Program, Keck School of Medicine of USC, 4650 Sunset Blvd, Los Angeles, California 90027 USA

**Keywords:** Congenital heart defects, Echocardiography, Access to health care, COVID-19

## Abstract

Prenatal diagnosis of critical congenital heart disease (CCHD) has improved over time, and previous studies have identified CCHD subtype and socioeconomic status as factors influencing rates of prenatal diagnosis. Our objective of this single-center study was to compare prenatal diagnosis rates of newborns with CCHD admitted for cardiac intervention from the COVID-19 pandemic period (March 2020 to March 2021) to the pre-pandemic period and identify factors associated with the lack of CCHD prenatal diagnosis. The overall rate of CCHD and rates of the various CCHD diagnoses were calculated and compared with historical data collection periods (2009–2012 and 2013–2016). Compared with the 2009–2012 pre-pandemic period, patients had 2.17 times higher odds of having a prenatal diagnosis of CCHD during the pandemic period controlling for lesion type (aOR = 2.17, 95% CI 1.36–3.48, *p* = 0.001). Single ventricle lesions (aOR 6.74 [4.64–9.80], *p* < 0.001) and outflow tract anomalies (aOR 2.20 [1.56–3.12], *p* < 0.001) had the highest odds of prenatal diagnosis compared with the remaining lesions. Patients with outflow tract anomalies had higher odds for prenatal detection in the pandemic period compared with during the 2009–2012 pre-pandemic period (aOR 2.01 [1.06–3.78], *p* = 0.031). In conclusion, prenatal detection of CCHD among newborns presenting for cardiac intervention appeared to have improved during the pandemic period.

## Introduction

Congenital heart disease (CHD) is the most common birth defect worldwide, affecting approximately 1.0-1.8% of all live births annually [1–4]. Critical congenital heart disease (CCHD), defined as CHD requiring surgical or catheter-based cardiac intervention within the first year of life, is specifically thought to affect 2.5 per 1000 live births annually in the United States (US) alone [2]. Infants born with CHD are at increased risk of postnatal complications, including severe acidosis, multi-organ injury, cardiac arrest, and death, with CHD being among the top eight leading causes of infant mortality in the first year of life worldwide [4, 5]. However, prenatal diagnosis has been associated with decreased mortality and severity of physiological sequelae, with improved outcomes due to the potential for planned delivery at a cardiac intervention center and prompt perinatal intervention [5, 6].

The rate of prenatal diagnosis of CCHD in the US has increased over the years, reaching 42% in 2012, given improved guidelines for second- and third-trimester ultrasound examination and fetal echocardiography referral [7]. However, several studies have shown significant variation in prenatal diagnosis rates across different geographic regions and populations. Certain socioeconomic factors, such as a lower median household income [8], public insurance [9], and living in a higher poverty and lower population density area [10], have been associated with lower rates of prenatal diagnosis of CHD and identified as potential areas for mitigation. However, it is currently unknown whether the coronavirus (COVID-19) pandemic has modified the impact of these factors or introduced new considerations.

With the introduction of California’s stay-at-home order in March 2020 and in the early pandemic period, rates of in-person healthcare utilization unrelated to COVID-19 declined, in part due to changing recommendations by healthcare professional groups [11, 12]. Within maternal-fetal medicine (MFM), statements by the International Society of Ultrasound in Obstetrics and Gynecology (ISUOG) [13], Society for Maternal-Fetal Medicine (SMFM) [14], and others [15], recommended reducing face-to-face contact when possible to limit the risk of disease transmission by increasing telehealth utilization and modifying routine ultrasound examination schedules based on local disease prevalence and reserving fetal echocardiography referrals for highest-risk patients. Similarly, the American Society of Echocardiography (ASE) recommended consolidating fetal echocardiogram exams with other MFM visits, utilizing telemedicine for counseling, and provided new indications to defer or delay cardiology referral [16, 17].

While these recommendations sought to balance COVID-19 restrictions with routine prenatal care, the modification of the traditional prenatal care schedule and management algorithm of pregnant women presented potential repercussions of the pandemic on prenatal diagnosis rates of CCHD, which rely on in-person ultrasound examinations and echocardiography referrals.

Given limited understanding of the impact of the pandemic on prenatal diagnosis of CCHD, and ongoing discourse regarding the factors affecting it, we sought to compare rates of prenatal diagnosis of CCHD during the COVID-19 pandemic with historical data. We hypothesized that prenatal diagnosis rates of CCHD had the potential to decrease during the pandemic period compared with historical rates, due to restrictions on non-COVID-19-related and in-person healthcare resulting from the stay-at-home order in March 2020. We also attempted to identify factors associated with a lack of prenatal diagnosis during the pandemic period.

## Methods

This was a single-center retrospective cohort study of newborns with CCHD admitted to Children’s Hospital Los Angeles (CHLA) for cardiac intervention for a 1-year period from March 19, 2020 (the date of California’s stay-at-home order) to March 18, 2021, referred to as the pandemic period. CCHD was defined as CHD requiring surgical or catheter-based cardiac intervention within the first 30 days of life. Patients with an isolated patent ductus arteriosus were excluded. We compared data from the pandemic period cohort with historical data from the periods of 2009–2012 and 2013–2016 of newborns admitted with CCHD at CHLA. The cohorts were divided at 2013 due to the updated 2013 American Institute of Ultrasound Medicine guidelines. This study was approved by our institutional review board with a waiver of informed consent.

For the pandemic period, patients were identified using the CHLA cardiothoracic surgery database, and electronic chart review was performed to collect fetal and maternal clinical and demographic data potentially associated with prenatal detection for the pandemic period. Maternal data included maternal age, maternal insurance type, and race/ethnicity. Insurance types were grouped into public (i.e., Medicaid, Medicaid health maintenance organization [HMO]) vs. commercial (i.e., preferred provider organization [PPO], HMO, integrated delivery system [IDS]). Race/ethnicity was classified based on patient self-identification to categories of Hispanic White, Non-Hispanic White, Hispanic Black, Non-Hispanic Black, Asian/Pacific Islander, or Other; categories were collapsed to examine associations with non-Hispanic White (yes/no).

For historical data retrieved for the pre-pandemic cohorts, patients were similarly identified using the CHLA cardiothoracic surgery database and cardiac diagnoses were extracted via electronic chart review, but maternal demographic data were not collected. For both the pandemic and pre-pandemic cohorts, each patient’s cardiac diagnosis was hierarchically classified based on their echocardiographic findings into the following mutually exclusive groups: (1) presence of a single ventricle (hypoplastic left heart syndrome, pulmonary atresia with intact ventricular septum, tricuspid stenosis/atresia, unbalanced atrioventricular canal defect); (2) outflow tract anomalies (Tetralogy of Fallot, double outlet right ventricle, transposition of great arteries with/without ventricular septal defect, truncus arteriosus, pulmonary atresia with ventricular septal defect and major aorto-pulmonary collateral arteries, aortic valve stenosis, double aortic arch, aorto-pulmonary window); and (3) any remaining lesions not yet categorized by the above categories (aortic arch hypoplasia/coarctation, interrupted aortic arch, total anomalous pulmonary venous return, isolated atrioventricular valve disease, balanced atrioventricular canal, ventricular septal defect). Total anomalous pulmonary venous return, arch obstruction, and ventricular septal defects are historically among the more difficult CCHD to diagnose prenatally [7].

Prenatal diagnosis rates for all CCHD and for each of the CHD lesion categories were described and compared using chi-square or Fisher’s exact testing as appropriate for the pre-pandemic vs. pandemic periods. A p-value < 0.05 was considered statistically significant. In an analysis of the entire population, a multiple logistic regression model for prenatal diagnosis was used to adjust for time period and CCHD lesion type. In a second set of logistic regression analyses, one for each lesion type, prenatal diagnosis was adjusted by time period. To identify risk factors for the lack of prenatal diagnosis for all CCHD during the pandemic period, bivariate analyses tested associations between prenatal diagnosis and insurance type, maternal age, patient race/ethnicity, and lesion type; a multiple logistic regression model was developed to test for association of these variables with prenatal diagnosis. Adjusted odds ratios (aOR) with 95% confidence intervals (CI) are reported.

## Results

In the pandemic period, 104 newborns were admitted to CHLA and underwent cardiac surgery within the first 30 days of life. 5 newborns with isolated patent ductus arteriosus were excluded, leaving 99 patients in the pandemic period cohort. From historical data, 398 newborns were identified who met CCHD inclusion criteria in the 2009–2012 pre-pandemic cohort and 393 newborns were identified in the 2013–2016 pre-pandemic cohort. Table 1 summarizes patient demographic factors and cardiac diagnoses by pre-pandemic versus pandemic period cohorts.

**Table 1:** Patient characteristics by time period

Patient characteristic	Pre-Pandemic Cohort 2009–2012 (*N* = 398)	Pre-Pandemic Cohort 2013–2016 (*N* = 393)	Pandemic Cohort (*N* = 99)	P-value
*Sex of newborn*				0.248
Ambiguous	0 (0%)	1 (0.3%)	0 (0%)	
Unknown	1 (0.3%)	0 (0%)	0 (0%)	
Female	176 (44.2%)	152 (38.7%)	50 (50.5%)	
Male	221 (55.5%)	240 (61.1%)	49 (49.5%)	
*Maternal age*			*N* = 96 30.9 ± 6.7	
*Insurance*				
Commercial			50 (50.5%)	
Public			49 (49.5%)	
*Race*			*N* = 97	
Hispanic White			46 (47.4%)	
Non-Hispanic White			26 (26.8%)	
Hispanic Black			1 (1.0%)	
Non-Hispanic Black			6 (6.2%)	
Asian/Pacific Islander			15 (15.5%)	
Other			3 (3.1%)	
Non-Hispanic White (yes)			26 (26.8%)	
Non-Hispanic White (no)			71 (73.2%)	
*CCHD Lesion Type*				
Single Ventricle^†^	138 (34.7%)	126 (32.1%)	17 (17.2%)	0.004
Any Outflow Tract Anomaly^‡^	149 (37.4%)	133 (33.8%)	53 (53.5%)	0.001
Remaining Lesions^§^	111 (27.9%)	134 (34.1%)	29 (29.3%)	0.158

^†^Hypoplastic left heart syndrome, pulmonary atresia with intact ventricular septum, tricuspid stenosis/atresia, unbalanced atrioventricular canal defect

^‡^Tetralogy of Fallot, double outlet right ventricle, transposition of great arteries with/without ventricular septal defect, truncus arteriosus, pulmonary atresia with ventricular septal defect and major aorto-pulmonary collateral arteries, aortic valve stenosis, double aortic arch, aorto-pulmonary window

^§^Aortic arch hypoplasia/coarctation, interrupted aortic arch, total anomalous pulmonary venous return, isolated atrioventricular valve disease, balanced atrioventricular canal, ventricular septal defect

Bivariate analysis for the 3 time periods was not statistically significant, with overall rates of prenatal diagnosis of 45.0% in 2009–2012, 45.8% in 2013–2016, and 57.6% in 2020–2021, *p* = 0.070 (Table 2). Prenatal diagnostic rates for each time period by lesion type can be seen in Table 2. After adjusting for lesion type, patients in the pandemic period had 2.17 times higher odds of having a prenatal diagnosis of CCHD compared with the 2009–2012 pre-pandemic cohort period (aOR = 2.17, 95% CI 1.36–3.48, *p* = 0.001). Across all time periods, the odds of a prenatal diagnosis of a single ventricle lesion were 6.74 times higher compared with the remaining lesion reference group (aOR = 6.74, 95% CI 4.64–9.80, *p* < 0.001) and the odds of having prenatal diagnosis of an outflow tract anomaly were 2.20 times higher than the remaining lesion reference group (aOR = 2.20, 95% CI 1.56–3.12, *p* < 0.001) (Table 3).

**Table 2:** Prevalence of prenatal diagnosis of patients undergoing cardiac surgery within first 30 days of life by time period

	Pre-Pandemic Cohort 2009–2012 (*N* = 398)	Pre-Pandemic Cohort 2013–2016 (*N* = 393)	Pandemic Cohort (*N* = 99)	P-value
Overall Prenatal Diagnosis: Yes (%)	179 of 398 (45.0%)	180 of 393 (45.8%)	57 of 99 (57.6%)	0.070
Single Ventricle Prenatal Diagnosis^†^: Yes (%)	93 of 138 (67.4%)	86 of 126 (68.3%)	16 of 17 (94.1%)	0.073
Any Outflow Tract Anomaly Diagnosis^‡^: Yes (%)	56 of 149 (37.6%)	64 of 133 (48.1%)	29 of 53 (54.7%)	0.054
Remaining Lesions^§^: Yes (%)	30 of 111 (27.0%)	30 of 134 (22.4%)	12 of 29 (41.4%)	0.106

^†^Hypoplastic left heart syndrome, pulmonary atresia with intact ventricular septum, tricuspid stenosis/atresia, unbalanced atrioventricular canal defect

^‡^Tetralogy of Fallot, double outlet right ventricle, transposition of great arteries with/without ventricular septal defect, truncus arteriosus, pulmonary atresia with ventricular septal defect and major aorto-pulmonary collateral arteries, aortic valve stenosis, double aortic arch, aorto-pulmonary window

^§^Aortic arch hypoplasia/coarctation, interrupted aortic arch, total anomalous pulmonary venous return, isolated atrioventricular valve disease, balanced atrioventricular canal, ventricular septal defect

**Table 3:** Multiple logistic regression model for prenatal diagnosis of critical congenital heart disease adjusting for time period and lesion type (c = 0.701)

Patient characteristic	Adjusted Odds Ratio (95% confidence interval)	P-value
*Time period*		
Pandemic cohort	2.17 (1.36–3.48)	0.001
Pre-pandemic cohort 2013–2016	1.13 (0.84–1.52)	0.433
Pre-pandemic cohort 2009–2012	reference	
*CCHD Lesion Type*		
Single Ventricle^†^	6.74 (4.64–9.80)	< 0.001
Any Outflow Tract Anomaly^‡^	2.20 (1.56–3.12)	< 0.001
Remaining Lesions^§^	reference	

^†^Hypoplastic left heart syndrome, pulmonary atresia with intact ventricular septum, tricuspid stenosis/atresia, unbalanced atrioventricular canal defect

^‡^Tetralogy of Fallot, double outlet right ventricle, transposition of great arteries with/without ventricular septal defect, truncus arteriosus, pulmonary atresia with ventricular septal defect and major aorto-pulmonary collateral arteries, aortic valve stenosis, double aortic arch, aorto-pulmonary window

^§^Aortic arch hypoplasia/coarctation, interrupted aortic arch, total anomalous pulmonary venous return, isolated atrioventricular valve disease, balanced atrioventricular canal, ventricular septal defect

Additionally, with simple logistic regression analysis, adjusting for time periods, patients with outflow tract anomalies had 2.01 times higher odds of having a prenatal diagnosis in the pandemic cohort than in the 2009–2012 pre-pandemic cohort period (aOR = 2.01, 95% CI 1.06–3.78, *p* = 0.031). While not statistically significant, patients with a single ventricle lesion had 7.74 higher odds of having a prenatal diagnosis in the pandemic period compared with the 2009–2012 pre-pandemic cohort period (aOR = 7.74, 95% CI 0.10-60.21, *p* = 0.051) (Table 4).

**Table 4:** Simple logistic regression models for prenatal diagnosis of critical congenital heart disease lesion type by time period

CCHD Lesion Type and Time Period	Adjusted Odds Ratio (95% confidence interval)	P-value
*Single Ventricle* ^*†*^ *(* *N* *= 281, c = 0.540)* *Time period*		
Pandemic cohort	7.74 (0.10-60.21)	0.051
Pre-pandemic cohort 2013–2016	1.04 (0.62–1.75)	0.881
Pre-pandemic cohort 2009–2012	reference	
*Any Outflow Tract Anomaly* ^*‡*^ *(* *N* *= 335, c = 0.570)* *Time period*		
Pandemic cohort	2.01 (1.06–3.78)	0.031
Pre-pandemic cohort 2013–2016	1.54 (0.96–2.48)	0.075
Pre-pandemic cohort 2009–2012	reference	
*Remaining Lesions* ^*§*^ *(* *N* *= 274, c = 0.565)* *Time period*		
Pandemic cohort	1.91 (0.82–4.46)	0.137
Pre-pandemic cohort 2013–2016	0.78 (0.44–1.40)	0.401
Pre-pandemic cohort 2009–2012	reference	

^†^Hypoplastic left heart syndrome, pulmonary atresia with intact ventricular septum, tricuspid stenosis/atresia, unbalanced atrioventricular canal defect

^‡^Tetralogy of Fallot, double outlet right ventricle, transposition of great arteries with/without ventricular septal defect, truncus arteriosus, pulmonary atresia with ventricular septal defect and major aorto-pulmonary collateral arteries, aortic valve stenosis, double aortic arch, aorto-pulmonary window

^§^Aortic arch hypoplasia/coarctation, interrupted aortic arch, total anomalous pulmonary venous return, isolated atrioventricular valve disease, balanced atrioventricular canal, ventricular septal defect

For the pandemic period cohort, patients with commercial insurance were more likely to have prenatal diagnosis of their CCHD compared with those with public insurance (35/57 [61.4%] vs. 15/42 [35.7%], *p* = 0.020) (Table 5). Prenatal diagnosis of CCHD in the pandemic period was not associated with race/ethnicity (*p* = 0.716) (Table 5). After adjusting for maternal age and Non-Hispanic White Race, and CCHD lesion type, patients with commercial insurance had 4.54 times higher odds of having a prenatal diagnosis of their CCHD compared with those with public insurance (aOR = 4.54, 95% CI 1.69–12.20, *p* = 0.003) (Table 6).

**Table 5:** Patient characteristics by prenatal diagnosis (yes/no) in pandemic cohort

Patient characteristic	Prenatal Diagnosis Yes (*N* = 57)	Prenatal Diagnosis No (*N* = 42)	P-value
*Insurance*			0.012
Commercial (vs. Public)	35 (61.4%)	15 (35.7%)	
*Race/ethnicity*	*N* = 56	*N* = 41	0.716
Hispanic White	29 (51.8%)	17 (41.5%)	
Non-Hispanic White	15 (26.8%)	11 (26.8%)	
Hispanic Black	0 (0%)	1 (2.4%)	
Non-Hispanic Black	3 (5.4%)	3 (7.3%)	
Asian/Pacific-Islander	8 (14.3%)	7 (17.1%)	
Other	1 (1.8%)	2 (4.9%)	
Race White	*N* = 56	*N* = 41	0.253
	44 (78.6%)	28 (68.3%)	
Race White Non-	*N* = 56	*N* = 41	1.000
Hispanic	15 (26.8%)	11 (26.8%)	
*CCHD Lesion Type*			
Single Ventricle^†^	16 (28.1%)	1 (2.4%)	< 0.001
Any Outflow Tract Anomaly^‡^	29 (50.9%)	24 (57.1%)	0.537
Remaining Lesions^§^	12 (21.1%)	17 (40.5%)	0.036

^†^Hypoplastic left heart syndrome, pulmonary atresia with intact ventricular septum, tricuspid stenosis/atresia, unbalanced atrioventricular canal defect

^‡^Tetralogy of Fallot, double outlet right ventricle, transposition of great arteries with/without ventricular septal defect, truncus arteriosus, pulmonary atresia with ventricular septal defect and major aorto-pulmonary collateral arteries, aortic valve stenosis, double aortic arch, aorto-pulmonary window

^§^Aortic arch hypoplasia/coarctation, interrupted aortic arch, total anomalous pulmonary venous return, isolated atrioventricular valve disease, balanced atrioventricular canal, ventricular septal defect

**Table 6:** Multiple logistic regression model for prenatal diagnosis of critical congenital heart disease (c = 0.783)

Patient characteristic	Adjusted Odds Ratio (95% confidence interval)	P-value
Commercial insurance	4.54 (1.69–12.20)	0.003
Maternal age	1.02 (0.94–1.09)	0.692
Race White Non-Hispanic	1.33 (0.46–3.88)	0.622
*CCHD Lesion Type*		
Single Ventricle^†^	30.41 (3.25–284.4)	0.003
Any Outflow Tract Anomaly^‡^	1.22 (0.43–3.51)	0.706
Remaining Lesions^§^	reference	

^†^Hypoplastic left heart syndrome, pulmonary atresia with intact ventricular septum, tricuspid stenosis/atresia, unbalanced atrioventricular canal defect

^‡^Tetralogy of Fallot, double outlet right ventricle, transposition of great arteries with/without ventricular septal defect, truncus arteriosus, pulmonary atresia with ventricular septal defect and major aorto-pulmonary collateral arteries, aortic valve stenosis, double aortic arch, aorto-pulmonary window

^§^Aortic arch hypoplasia/coarctation, interrupted aortic arch, total anomalous pulmonary venous return, isolated atrioventricular valve disease, balanced atrioventricular canal, ventricular septal defect

## Discussion

Our study found that overall rates of prenatal diagnosis of CCHD in patients admitted for cardiac intervention appear to have improved over the 2020–2021 COVID-19 pandemic period. This was contrary to our initial hypothesis that rates of prenatal diagnosis would decrease in the face of restrictions on non-COVID-19-related and in-person healthcare due to the stay-at-home order in March 2020, as well as practice parameter recommendations by the ISUOG [13], SMFM [14], and ASE [16, 17], limiting face-to-face visits and frequency of ultrasound examinations.

While we had anticipated that such conditions would impact the ability of patients to receive ultrasound examinations and attend echocardiography referral appointments, leading to decreased quality of prenatal care and missed fetal cardiac screening opportunities, we postulate that this continued uptrend in prenatal diagnosis rates may have been augmented by successful COVID-19 accommodations within obstetric care as well as more comprehensive screening recommendations by multiple society guidelines. Several recent studies of prenatal care during the pandemic have suggested equal or even improved prenatal care utilization, including increased rates of initiation of prenatal care in the first trimester, unchanged numbers of total prenatal care visits, and equal-to-increased number of dating and anatomy ultrasound examinations [18–20]. One preliminary study that examined prenatal diagnosis of CHD at a single-center fetal therapy clinic that had implemented increased use of telehealth specifically found that patients had a similar gestational age at initial diagnosis of CHD, an earlier gestational age at referral and first visit, and longer pediatric cardiology counseling [20]. This was postulated to be due to the ability for patients to develop more comprehensive and individualized care plans with their care team via the flexibility of telemedicine appointments.

If the improvement in prenatal diagnosis were truly independently associated with the pandemic, then our findings suggest that despite COVID-19 restrictions and changes in the timing of routine prenatal care, providers were able to ensure that patients still met vital checkpoints during their pregnancies, resulting in adequate referral rates and a rise in CCHD prenatal diagnosis rates as seen in our study. However, it is also possible that sites that were under-resourced or unable to effectively transition care to accommodate pandemic restrictions, may have faced disparate rates of care utilization.

Although the continued increase in prenatal diagnosis rates of CCHD during the first year of the pandemic is reassuring, given the correlation between prenatal diagnosis and reduced mortality and morbidity [5, 6], future studies are warranted to evaluate whether this upward trend in prenatal diagnosis rates continues to increase through the second year of the pandemic and now in the post-pandemic period, with the expiration of the COVID-19 public health emergency on May 11, 2023. Given that pregnancy is a long-term process, the presence or lack thereof of a CCHD diagnosis during the pandemic may have been dependent on the gestational age of the fetus at the time the stay-at-home order was put in place. Those in later stages of their pregnancy when the pandemic started may have benefitted from in-person services earlier in their prenatal care that were conducive to obtaining a prenatal diagnosis during the pandemic.

It is also noteworthy that prenatal diagnosis rates of outflow tract lesions had significantly higher odds during the pandemic compared with the 2009–2012 cohort, while single ventricle lesions did not show a statistically significant increase, which may have been limited by sample size. As single-ventricle lesions may be diagnosed with the standard 4-chamber view, compared to outflow tract lesions that are better visualized or can only be visualized with additional outflow tract views, this suggests that diagnosis rates of lesions requiring more advanced fetal echocardiography may have improved over time. Previous studies identified decreased rates of prenatal diagnosis of CHD lesions requiring additional views for lesion visualization, such as outflow tract and/or aortic arch views, especially in rural communities that are less likely to obtain further imaging training [7, 8, 10, 21]. Updated practice guidelines in 2013 and 2018 for standard obstetric ultrasound examinations in the US mandated outflow tract views, as well as three-vessel and three-vessel trachea view in the routine second- and third-trimester ultrasound examinations [22, 23], while updated guidelines in 2020 for fetal echocardiography required additional assessment of pulmonary venous anatomy and flow, as well as cardiac biometry of the heart valves [24]. The improvement in detection of outflow tract lesions in the setting of these updated guidelines could represent successful adoption and implementation of these guidelines but warrants further examination.

In our subset analysis of fetal and maternal demographic data of the pandemic cohort, we found an association between medical insurance type and rates of prenatal diagnosis, with privately-insured patients being significantly more likely to receive a prenatal diagnosis of CCHD during the pandemic. Peiris et al., previously identified private medical insurance as the single strongest risk factor of prenatal diagnosis of CCHD, even more so than overall socioeconomic position based on education, occupation, and income [9]. In our model, single ventricle anatomy had the strongest association with prenatal diagnosis of CCHD in the pandemic cohort.

Lastly, our study did not find significant associations between race/ethnicity and rates of prenatal diagnosis of CCHD. This differs from previous studies that have shown Hispanic ethnicity to be significantly associated with decreased rates of prenatal diagnosis [21, 25] but agrees with Peiris et al., who did not find a relationship between race/ethnicity and prenatal diagnosis [9]. Considering that certain racial and ethnic groups have historically decreased access to healthcare, it is interesting that race/ethnicity did not result in a significant effect in our study. This discrepancy could be attributed to insurance patterns and geographical variation within the greater Los Angeles area, such as a relatively high proportion of Hispanic residents, as demonstrated by our patient population being 48% Hispanic. It is also possible that there are other socioeconomic factors shared across various race/ethnicity subgroups, such as the aforementioned insurance types, income levels, or geographic locations, which may be stronger predictors than the racial/ethnic subgroup itself.

## Limitations

As our study cohort was small, particularly the pandemic cohort, and limited to patients in a single-center database in Los Angeles, the generalizability of our results to other centers and regions is limited. Future studies conducted at a variety of fetal cardiac centers throughout the US are needed to corroborate our findings of increased prenatal diagnosis rates during the pandemic period. Secondly, the denominator for this study only includes patients who underwent cardiac surgery at CHLA within 30 days of life. The study also did not include CCHD patients that underwent catheterization-based interventions. Any patients who were not admitted for surgery at CHLA by this time, for any reason, such as death, intra-uterine fetal demise, transfer to a different surgical center from birthing hospital, were not included. For the purposes of this study, we have assumed that there was no differential loss or gain of CCHD patients in any of the time periods, i.e., that proportionally, losses and gains of CCHD patients remained the same. However, undocumented changes in referral patterns for CCHD may have occurred during the pandemic and contributed to increased prenatal diagnosis rates at CHLA resulting in selection bias. Finally, our study only included fetal and maternal demographic data from the pandemic cohort. Without similar data from the historical cohort, we cannot assess how the effect of insurance type and race/ethnicity on prenatal CCHD could have changed over time. Future studies may examine whether race/ethnicity, insurance type, and other socioeconomic factors were associated with prenatal diagnosis in the past.

## Conclusion

Prenatal detection of CCHD has continued to improve significantly over the past decade even during the pandemic period, despite COVID-19 restrictions and practice parameter recommendations that limited the frequency of face-to-face visits and access to ultrasound examinations by fetal cardiologists. Increased prenatal diagnosis was particularly notable in the outflow tract anomaly lesion subtype of CCHD.

## Data Availability

The data that support the findings of this study are available from the corresponding author upon request.

## References

[1] Tsao CW, Aday AW, Almarzooq ZI (2022). Heart Disease and Stroke Statistics-2022 update: a Report from the American Heart Association. Circulation.

[2] Hoffman JI, Kaplan S (2002). The incidence of congenital heart disease. J Am Coll Cardiol.

[3] van der Linde D, Konings EE, Slager MA (2011). Birth prevalence of congenital heart disease worldwide: a systematic review and meta-analysis. J Am Coll Cardiol.

[4] GBD 2017 Congenital Heart Disease Collaborators (2020). Global, regional, and national burden of congenital heart disease, 1990–2017: a systematic analysis for the global burden of Disease Study 2017. Lancet Child Adolesc Health.

[5] Schultz AH, Localio AR, Clark BJ (2008). Epidemiologic features of the presentation of critical congenital heart disease: implications for screening. Pediatrics.

[6] Cloete E, Bloomfield FH, Sadler L (2019). Antenatal detection of treatable critical congenital heart disease is associated with lower morbidity and mortality. J Pediatr.

[7] Quartermain MD, Pasquali SK, Hill KD (2015). Variation in prenatal diagnosis of congenital heart disease in infants. Pediatrics.

[8] Campbell MJ, Lorch S, Rychik J (2021). Socioeconomic barriers to prenatal diagnosis of critical congenital heart disease. Prenat Diagn.

[9] Peiris V, Singh TP, Tworetzky W (2009). Association of socioeconomic position and medical insurance with fetal diagnosis of critical congenital heart disease. Circ Cardiovasc Qual Outcomes.

[10] Hill GD, Block JR, Tanem JB, Frommelt MA (2015). Disparities in the prenatal detection of critical congenital heart disease. Prenat Diagn.

[11] Kim Y, Gordon A, Rowerdink K (2022). The impact of the COVID-19 pandemic on health care utilization among insured individuals with common chronic conditions. Med Care.

[12] Xu S, Glenn S, Sy L (2021). Impact of the COVID-19 pandemic on health care utilization in a large integrated health care system: retrospective cohort study. J Med Internet Res.

[13] Abu-Rustum RS, Akolekar R, Sotiriadis A (2020). ISUOG Consensus Statement on organization of routine and specialist obstetric ultrasound services in context of COVID-19. Ultrasound Obstet Gyencol.

[14] The Society for Maternal-Fetal Medicine (2020) COVID-19 Ultrasound Clinical Practice Suggestions https://s3.amazonaws.com/cdn.smfm.org/media/2550/Ultrasound_Covid19_Suggestions_10-20-20_(final).pdf

[15] Boelig RC, Saccone G, Bellussi F, Berghella V (2020). MFM guidance for COVID-19. Am J Obstet Gynecol MFM.

[16] Kirkpatrick JN, Mitchell C, Taub C (2020). ASE Statement on protection of patients and echocardiography service providers during the 2019 novel coronavirus outbreak: endorsed by the American College of Cardiology. J Am Coll Cardiol.

[17] Barker PCA, Lewin MB, Donofrio MT (2020). Specific considerations for pediatric, fetal, and congenital heart disease patients and echocardiography service providers during the 2019 novel coronavirus outbreak: Council on Pediatric and congenital heart Disease supplement to the Statement of the American Society of Echocardiography: endorsed by the Society of Pediatric Echocardiography and the fetal Heart Society. J Am Soc Echocardiogr.

[18] Boguslawski SM, Joseph NT, Stanhope KK (2022). Impact of the COVID-19 pandemic on prenatal care utilization at a public hospital. Am J Perinatol.

[19] Kern-Goldberger AR, Sheils NE, Ventura MEM (2023). Patterns of prenatal care delivery and obstetric outcomes before and during the COVID-19 pandemic. Am J Perinatol.

[20] Ronai C, Canty E, Kim A (2021). Abstract 11221: counseling in the time of COVID – prenatal diagnosis of congenital heart disease during a pandemic. Circulation.

[21] Pinto NM, Keenan HT, Minich LL (2012). Barriers to prenatal detection of congenital heart disease: a population-based study. Ultrasound Obstet Gynecol.

[22] American Institute of Ultrasound in Medicine (2013). AIUM practice guideline for the performance of obstetric ultrasound examinations. J Ultrasound Med.

[23] AIUM-ACR-ACOG-SMFM-SRU (2018). Practice parameter for the performance of Standard Diagnostic Obstetric Ultrasound examinations. J Ultrasound Med.

[24] AIUM Practice Parameter for the Performance of Fetal Echocardiography (2020). J Ultrasound Med.

[25] Ailes EC, Gilboa SM, Riehle-Colarusso T, National Birth Defects Prevention Study (2014). Prenatal diagnosis of nonsyndromic congenital heart defects. Prenat Diagn.

